# Simulation of two electrode reactions coupled by the chemical reaction

**DOI:** 10.55730/1300-0527.3429

**Published:** 2022-04-11

**Authors:** Milivoj LOVRIĆ

**Affiliations:** Divkovićeva 13, Zagreb, Croatia

**Keywords:** ECE mechanism, rotating disk electrode, staircase cyclic voltammetry, simulation, polarographic wave

## Abstract

A model of an ECE mechanism consisting of two reversible electrode reactions coupled by the kinetically controlled, second order, reversible chemical reaction is developed for the staircase cyclic voltammetry on the rotating disk electrode. The relationship between the limiting current of the second wave and the rate constant of chemical reaction is investigated. The concentration of electroinactive component of the forward reaction influences the half-wave potential of the first wave and the limiting current of the second wave. The responses of the mechanisms with reversible and irreversible chemical reactions are compared.

## 1. Introduction

In organic electrolytes, electrode reactions of many compounds, such as catechols [1] and quinones [2], consist of two successive electron transfers connected by the transfer of protons [3–5]. These reactions are examples of the ECE mechanism [6–12] and the nine-member square scheme [13–15]. They were observed in electroreduction of p-nitrosophenol [16, 17], benzenesulfonyl floride [18, 19] and hexacyanochromate(III) [20] and in electrooxidation of tocopherols [21], methylcatechol [22, 23] and dopamine [24]. The theory of this mechanism is developed for polarography [8, 25–29], cyclic voltammetry [20, 30–33], square wave voltammetry [34–38], rotating disk measurements [39, 40] and the surface reactions in protein film voltammetry [41, 42]. The steady state responses on the rotating disk were calculated for totally irreversible chemical reactions and in this paper the investigation is extended to kinetically controlled, second order, reversible chemical reactions. The role of the electroinactive component of the reaction is analysed.

## 2. Model

It is assumed that an electrolytic solution contains dissolved reactant of the first electrode reaction and an electroinactive substance Y that cannot react with the mentioned reactant. The first electron transfer is fast and reversible electrooxidation. Its product cannot participate in the second electron transfer, but it can react with the substance Y to produce a compound that is stable at the given potential, but can be electrooxidized at higher potentials. Both chemical reaction and the second electrode reaction are reversible and the latter is fast. This mechanism can be represented by the following chemical equations:


(1) $$\text{A}↔\text{B}+{\text{e}}^{-}$$

(2) B+Y↔G

(3) $$\text{G}↔\text{H}+{\text{e}}^{-}$$

On the rotating disk electrode, the mass transfer and currents are defined by the following system of differential equations and the initial and boundary conditions:


(4) $$\frac{\partial c_{A}}{\partial t}=D\frac{\partial^{2}c_{A}}{\partial x^{2}}-v\frac{\partial c_{A}}{\partial x}$$

(5) $$\frac{\partial c_{B}}{\partial t}=D\frac{\partial^{2}c_{B}}{\partial x^{2}}-v\frac{\partial c_{B}}{\partial x}-k_{f}c_{B}c_{Y}+k_{b}c_{G}$$

(6) $$\frac{\partial c_{Y}}{\partial t}=D\frac{\partial^{2}c_{Y}}{\partial x^{2}}-v\frac{\partial c_{Y}}{\partial x}-k_{f}c_{B}c_{Y}+k_{b}c_{G}$$

(7) $$\frac{\partial c_{G}}{\partial t}=D\frac{\partial^{2}c_{G}}{\partial x^{2}}-v\frac{\partial c_{G}}{\partial x}+k_{f}c_{B}c_{Y}-k_{b}c_{G}$$

(8) $$\frac{\partial c_{H}}{\partial t}=D\frac{\partial^{2}c_{H}}{\partial x^{2}}-v\frac{\partial c_{H}}{\partial x}$$

(9) $$t=0,x\geq 0:c_{A}=c_{A}^{*},c_{B}=0,c_{Y}=c_{Y}^{*},c_{G}=0,c_{H}=0$$

(10) $$t>0,x\to \infty :c_{A}=c_{A}^{*},c_{B}\to 0,c_{Y}=c_{Y}^{*},c_{G}\to 0,c_{H}\to 0$$

(11) $$x=0:\;\;\;c_{B,x=0}-c_{A,x=0}\;exp\;\left(\frac{F}{RT}(E-E_{1}^{0})\right)$$

(12) $$c_{H,x=0}=c_{G,x=0}\;exp\;\left(\frac{F}{RT}(E-E_{2}^{0})\right)$$

(13) $$D\;{\left(\frac{\partial c_{A}}{\partial x}\right)}_{x=0}=\frac{I_{1}}{FS}$$

(14) $$D\;{\left(\frac{\partial c_{B}}{\partial x}\right)}_{x=0}=-\frac{I_{1}}{FS}$$

(15) $${\left(\frac{\partial c_{Y}}{\partial x}\right)}_{x=0}=0$$

(16) $$D\;{\left(\frac{\partial c_{G}}{\partial x}\right)}_{x=0}=\frac{I_{2}}{FS}$$

(17) $$D\;{\left(\frac{\partial c_{H}}{\partial x}\right)}_{x=0}=-\frac{I_{2}}{FS}$$

(18) $$v=-0.510\;\omega^{3/2}\nu^{-1/2}x^{2}$$

(19) $$K=\frac{k_{f}}{k_{b}}.$$

The meanings of all symbols are reported in Table. Equations (4)–(8) were solved by the finite difference method [43]. The current was calculated for the staircase cyclic voltammetry. The dimensionless current is defined by the following equations:


(20) $$\Phi =(I_{1}+I_{2})\delta_{ss}/FSc_{A}^{*}D$$

(21) $$\delta_{ss}=1.61\;D^{\frac{1}{3}}\nu^{\frac{1}{6}}\omega^{-\frac{1}{2}}.$$

The following parameters were not changed: *D* = 10^−5^ cm^2^/s, *v* = 10^−2^ cm^2^/s, Δ*t* = 10^−5^ s, $\frac{D\Delta t}{\Delta x^{2}}=0.2$,Δ*E* = 1 mV and *τ* = 10 ms.

## 3. Results and discussion

**Figure 1:**
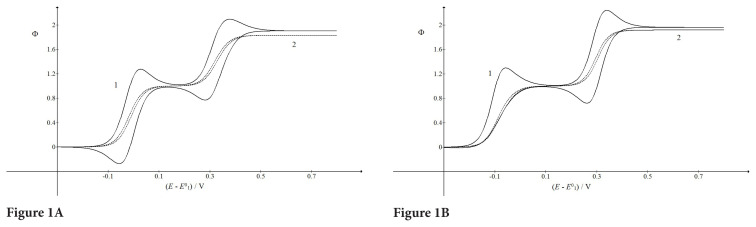
Dimensionless cyclic staircase voltammograms of the ECE mechanism on the rotating disk electrode under the transient (full line) and near steady-state (broken line) conditions. $E_{2}^{0}-E_{1}^{0}=0.3\;\text{V}$, Delta*E*/*τ* = 0.1 V/s, $c_{Y}^{*}/c_{A}^{*}=1,\;k_{f}\;c_{A}^{*}=10^{4}{\text{s}}^{-1},\;Kc_{A}^{*}=1\;(\text{A})$ and 10^3^ (B) and *ω*/rad s^−1^ = 4*π* (1) and 40*π* (2)

On the rotating disk electrode, the cyclic staircase voltammograms of the ECE mechanism with stable intermediates depend on the scan rate, the electrode rotation rate and the kinetics of chemical reaction. Some examples are shown in Figure 1. They were calculated assuming that the concentration of substance Y can be freely changed and adjusted to the concentration of the reactant A. If the rate constants of chemical reaction are very high and equal ( $Kc_{A}^{*}=1$) and the rotation rate is low, the response is characterized by two maxima and two minima at 0.027 V, 0.378 V, 0.281 V, and −0.057 V vs. $E_{1}^{0}$, respectively. Considering that $E_{2}^{0}-E_{1}^{0}=0.3\;\text{V}$, the two parts of the response are well separated. The last minimum at −0.057 V is important because it shows that very fast chemical reaction is transforming the second reactant G into the first product B in the reverse branch of voltammograms. This minimum is the evidence that the whole ECE mechanism is close to the equilibrium. The absence of this minimum that can be noted in Figure 1B shows that chemical reaction is irreversible. It is a consequence of very high equilibrium constant and very low backward rate constant that cannot produce the first product B in the reverse branch. The potentials of two maxima and the minimum are: −0.058 V, 0.340 V, and 0.261 V, respectively. The potential of the first maximum is 85 mV lower than in Figure 1A because the chemical reaction is consuming the product of the first electrode reaction and decreasing the formal potential of the first electron transfer [33].

At higher rotation rates, the maxima and minima disappear and the response acquires the form of polarogram. The dimensionless limiting current of the first wave is equal to unit at 0.15 V vs. $E_{1}^{0}$. This is the base line for the measurement of limiting currents of the second wave. In Figure 1A the half-wave potentials are −0.019 V and 0.321 V in the anodic branch and −0.010 V and 0.330 V in the cathodic branch. This is because this response corresponds to the near steady state conditions. Under strict steady state both branches are overlapped. If $Kc_{A}^{*}=10^{3}$ the first wave appears at −0.082 V because of irreversibility of chemical reaction.

**Figure 2:**
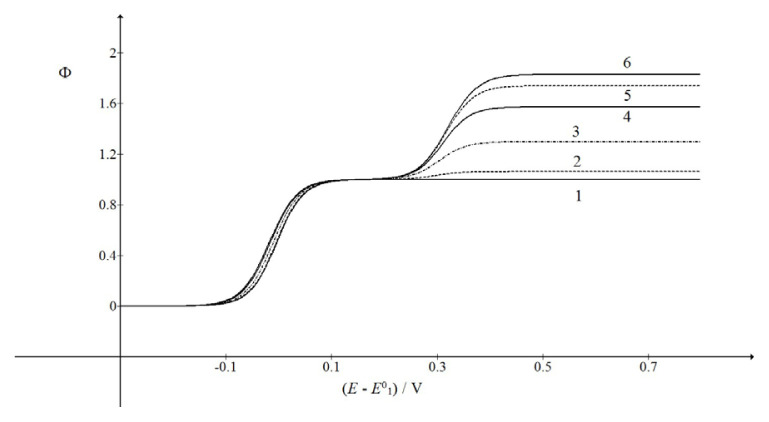
Dependence of the second wave on the product of the forward rate constant of chemical reaction and the bulk concentration of the reactant A. $Kc_{A}^{*}=1$, *ω* = 40*π* rad/s and $k_{f}\;c_{A}^{*}/{\text{s}}^{-1}=0\;(1)$, 1 (2), 10 (3), 100 (4), 1000 (5) and 10000 (6). All other data are as in Figure 1.

**Figure 3:**
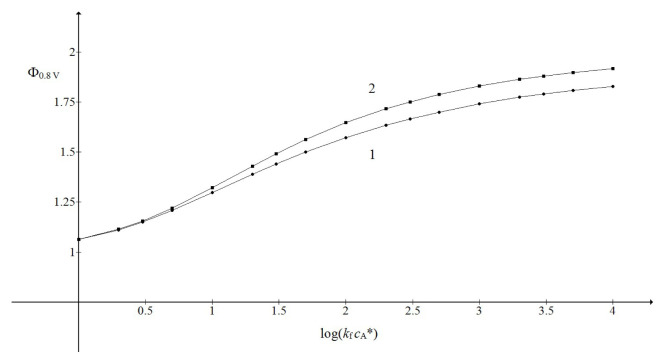
Dependence of the limiting current of the second wave on the logarithm of the product $k_{f}\;c_{A}^{*}$. $Kc_{A}^{*}=1$ (1) and 10^3^ (2). All other data are as in Figure 2.

Limiting currents of the second wave depend on the normalized forward rate constant of the chemical reaction. This is shown in Figure 2. The half-wave potentials of the second wave are changing from 0.298 V vs. $E_{1}^{0}\;(k_{f}\;c_{A}^{*}=1\;{\text{s}}^{-1})$ to 0.321 V (curve 6) and those of the first wave from −0.004 V (1) to −0.019 V (6). These opposite trends are caused by the increasing rate of chemical reaction that creates a combination of EC and CE mechanisms. Furthermore, Figure 1A shows that the current at 0.8 V decreases with the increasing rotation rate. This is because the time that the product B spends near the electrode surface is longer if the rotation rate is slower. The limiting currents of the second wave depend on the logarithm of the product $k_{f}\;c_{A}^{*}$ in a sigmoidal manner, as can be seen in Figure 3. This figure also shows that the limiting currents of irreversible chemical reaction are higher than those of reversible one. Obviously, the backward component of the reaction diminishes its net gain. The relationship of this type can be represented by the general function [44]:


(22) $$\Phi =1+{\left(k_{f}\;c_{A}^{*}\right)}^{p}/\left[{\left(k_{f}\;c_{A}^{*}\right)}^{p}+{\left(k_{f}\;c_{A}^{*}\right)}_{1/2}^{p}\right].$$

**Figure 4:**
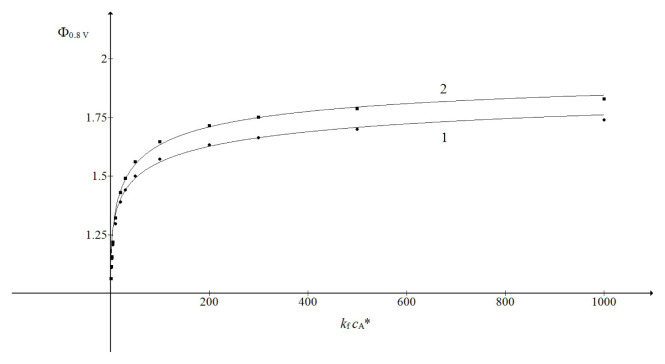
Dependence of the limiting currents of the second wave (the symbols) on the product $k_{f}\;c_{A}^{*}$. $Kc_{A}^{*}=1$ (1) and 10^3^ (2). The lines 1 and 2 are calculated by Eq. (22) using parameters *p* = 0.4 and ${\left(k_{f}\;c_{A}^{*}\right)}_{1/2}=54.54\;(1)$ and *p* = 0.5 and ${\left(k_{f}\;c_{A}^{*}\right)}_{1/2}=33.34\;(2)$. All other data are as in Figure 2.

Figure 4 shows that this representation is suitable for the limited range of the argument. The parameters *p* and ${\left(k_{f}\;c_{A}^{*}\right)}_{1/2}$ can be determined by the logarithmic analysis:


(23) $$log\Psi =p\;log(k_{f}\;c_{A}^{*})-p\;log{\left(k_{f}\;c_{A}^{*}\right)}_{1/2}$$

(24) Ψ=(Φ-1)/[1-(Φ-1)].

Firstly the function Ψ is calculated and then the parameters are measured from the relationship between *log*Ψ and $log(k_{f}\;c_{A}^{*})$. For instance, the limiting currents shown by the curve 2 in Figure 3 when transformed by Eq. (24) gave a set of log values that depended linearly on the corresponding $\text{log}(k_{f}\;c_{A}^{*})$ arguments, with the slope 0.5 and the intercept 5.8 (not shown). The curves 1 and 2 in Figure 4 are defined by the following functions:


(25) $$\Phi =1+{\left(k_{f}\;c_{A}^{*}\right)}^{0.4}/\left[{\left(k_{f}\;c_{A}^{*}\right)}^{0.4}+54.54^{0.4}\right]$$

(26) $$\Phi =1+\sqrt{k_{f}\;c_{A}^{*}}/[\sqrt{k_{f}\;c_{A}^{*}}+\sqrt{33.34}].$$

This procedure is useful for the transformation of the set of discrete data into a continuous function. By the variation of concentrations of A and Y, keeping them equal, one can estimate the parameter *p* from the gradient $\partial log\Psi /\partial logc_{A}^{*}$. If the limiting current of the second wave is one half of the first limiting current and ω=40π rad/s, then logΨ=0 and $logk_{f}=log44-log{\left(c_{A}^{*}\right)}_{1/2}$. The number 44 is an average of ${\left(k_{f}\;c_{A}^{*}\right)}_{1/2}$ values corresponding to reversible and irreversible chemical reactions. However, this estimation is rough and requires that the concentration of Y can be freely changed, which does not have to be fulfilled.

**Figure 5:**
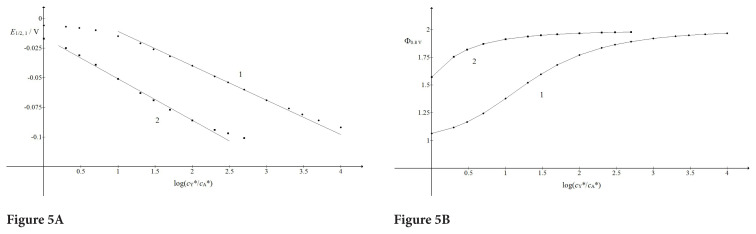
Dependence of the half-wave potential of the first wave (A) and the limiting current of the second wave (B) on the logarithm of the ratio between bulk concentrations of the substance Y and the reactant A; $k_{f}\;c_{A}^{*}/{\text{s}}^{-1}=1\;(1)$ and 100 (2). The straight lines 1 and 2 are defined by Eqs. (27) and (28), respectively. All other data are as in Figure 2.

The variation of the bulk concentration of the substance Y influences both waves of the ECE response. The limiting current of the first wave is independent of but the half-wave potential of this wave decreases significantly with the increasing of this concentration. Regarding the second wave, its limiting current is influenced mostly, while *E*_1/2,2_ is less affected. This is shown in Figure 5 for the reversible chemical reaction. The compound Y contributes to the forward rate of chemical reaction and its influence depends on the product $k_{f}\;c_{A}^{*}$. The relationship between *E*_1/2,1_ and $\text{log }(c_{Y}^{*}/c_{A}^{*})$ is sigmoidal and in the vicinity of inflexion points it can be approximated by the straight lines:


(27) $$E_{1/2,1}-E_{1}^{0}=-0.029\;\text{log }(c_{Y}^{*}/c_{A}^{*})+0.018\;\text{V}$$

(28) $$E_{1/2,1}-E_{1}^{0}=-0.035\;\text{log }(c_{Y}^{*}/c_{A}^{*})-0.016\;\text{V}\text{.}$$

If the dimensionless equilibrium constant is 10^3^ this relationship is very similar and the straight lines are:


(29) $$E_{1/2,1}-E_{1}^{0}=-0.028\;\text{log }(c_{Y}^{*}/c_{A}^{*})+0.015\;\text{V}$$

(30) $$E_{1/2,1}-E_{1}^{0}=-0.024\;\text{log }(c_{Y}^{*}/c_{A}^{*})-0.044\;\text{V}\text{.}$$

They apply for $k_{f}\;c_{A}^{*}$ 1 s^−1^ and 100 s^−1^, respectively. The limiting currents of the second wave are the sigmoidal function of $\text{log }(c_{Y}^{*}/c_{A}^{*})$ if $k_{f}\;c_{A}^{*}$ 1 s^−1^, but this relationship is only an upper fraction of the sigmoidal curve if 100 s^−1^. This is because these two arguments add up giving the joint effect. If $Kc_{A}^{*}=10^{3}$ the second half-wave potential is independent of $c_{Y}^{*}$, but if $Kc_{A}^{*}=1$ it changes from the values reported in Figure 2 to 0.296 V vs. $E_{1}^{0}$, which is the potential that corresponds to the irreversible chemical reaction. This change occurs within the interval $1<c_{Y}^{*}/c_{A}^{*}<50$.

**Figure 6:**
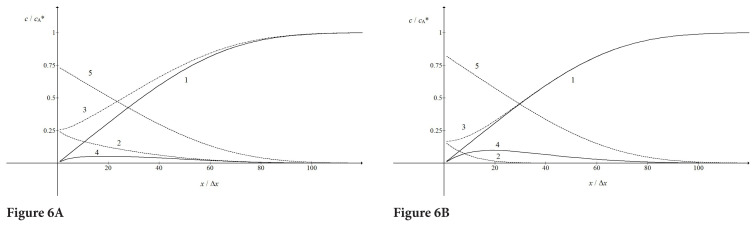
Distribution of the reactant A (1), the product B (2), the compound Y (3), the second reactant G (4) and the final product H (5) in the diffusion layer at 0.8 V vs. $E_{1}^{0}$; ω= 40 rad/s, $C_{Y}^{*}/C_{A}^{*}=1,k_{f}\;c_{A}^{*}10^{3}{\text{s}}^{-1}$ and $Kc_{A}^{*}=1\;(\text{A})$ and 10^3^ (B). All other data are as in Figure 1.

The difference in limiting currents of the second wave between the ECE mechanisms with reversible and irreversible chemical reactions can be explained by the inspection of distribution of chemical species in the diffusion layer that is shown in Figure 6. One can notice that the dimensionless concentrations of B, Y and G are particularly sensitive to the dimensionless equilibrium constant of chemical reaction. If the reaction is reversible the concentration of Y at the electrode surface is 0.26 and the maximum concentration of G is 0.05, but under the influence of irreversible reaction these concentrations change to 0.17 and 0.1, respectively. Consequently, the gradient of H is steeper if the reaction is irreversible and the current is higher.

The models of ECE mechanism in the voltammetry on stationary macroelectrodes give qualitative descriptions of the responses because the transient currents consist of the diffusion and kinetic components [25, 33, 34, 38]. Under steady-state conditions on microelectrodes [6] and rotating disk electrodes [40] the diffusion component can be controlled and the kinetic contribution can be quantified. In this paper, it is shown that the kinetic current satisfies a general equation (22) and that the rate constant of chemical reaction can be estimated under certain conditions.

## 4. Conclusion

The difference between ECE mechanisms with the reversible and irreversible chemical reactions originates from the influence of the backward rate constant on the net gain of the reaction. If the reaction is of the second order, its forward rate depends on the component Y and the limiting current of the second wave becomes higher if the concentration of Y is increased. The rate constant can be estimated from the relationship between currents and the concentrations of the reactant of the first electrode reaction.

## Figures and Tables

**Table:** Meanings of symbols.

Symbol	Meaning
*c* *_Z_*	Concentration of species Z
$c_{A}^{*},c_{Y}^{*}$	Bulk concentrations of species A and Y
*D*	Diffusion coefficient
*δ*	Diffusion layer thickness
Δ***E***	Potential increment
Δ***t***	Time increment
**Δ** ***x***	Space increment
*E*	Potential
$E_{1}^{0},E_{2}^{0}$	Standard potentials
*F*	Faraday constant
*I* *_1_* *,I* *_2_*	Currents
ν	Kinematic viscosity
*k_f_*, *k_b_*	Rate constants of chemical reaction
*K*	Equilibrium constant of chemical reaction
*ω*	Rotation rate
*R*	Gas constant
*S*	Electrode surface area
*T*	Time
*T*	Temperature
*τ*	Step duration
*V*	Flow rate of solution

## Data Availability

All relevant data are available on demand.

## References

[1] Lin Q Li Q Batchelor-McAuley C Compton RG Two-electron, two-proton oxidation of catechol: kinetics and apparent catalysis The Journal of Physical Chemistry C 2015 119 3 1489 1495 10.1021/jp511414b

[2] Gulaboski R Markovski V Jihe Z Redox chemistry of coenzyme Q – a short overview of the voltammetric features Journal of Solid State Electrochemistry 2016 20 12 3229 3238 10.1007/s10008-3230-7

[3] Wawzonek S Berkey R Blaha EW Runner ME Polarographic studies in acetonitrile and dimethylformamide. III. Behavior of quinones and hydroquinones Journal of the Electrochemical Society 1956 103 8 456 459

[4] Kolthoff IM Reddy TB Polarography and voltammetry in dimethylsulfoxide Journal of the Electrochemical Society 1961 108 10 980 984

[5] Peover ME A polarographic investigation into the redox behaviour of quinones: the roles of electron affinity and solvent Journal of the Chemical Society 1962 4540 4549

[6] Molina A Laborda E Gomez-Gil J Martinez-Ortiz F Compton RG A comprehensive voltammetric characterisation of ECE processes Electrochimica Acta 2016 195 230 245 10.1016/elecacta.2016.01.120

[7] Feldberg SW Nuances of the ECE mechanism. III. Effects of homogeneous redox equilibrium in cyclic voltammetry The Journal of Physical Chemistry 1971 75 15 2377 2380 10.1021/j100684a025

[8] Amatore C Saveant JM Do ECE mechanisms occur in conditions where they could be characterized by electrochemical kinetic techniques? Journal of Electroanalytical Chemistry and Interfacial Electrochemistry 1978 86 1 227 232 10.1016/S0022-0728(78)80371-4

[9] Palys MJ Bos M van der Linden WE Automatic polarographic elucidation of electrode mechanisms by means of a knowledge-based systems: Part 3. Mechanisms ECE, EE and mechanisms involving adsorption Analytica Chimica Acta 1993 283 2 811 829 10.1016/0003-2670(93)85296-V

[10] Sanecki PT Amatore C Skital PM The problem of the accuracy of electrochemical kinetic parameter determination for the ECE reaction mechanism Journal of Electroanalytical Chemistry 2003 546 109 121 10.1016/S0022-0728(03)00138-4

[11] Sanecki PT Skital PM The mathematical models of kinetics of the E, EC, ECE, ECEC, ECE-ECE and ECEC-ECEC processes with potential-dependent transfer coefficient as a rationale of isoalpha points Electrochimica Acta 2008 53 26 7711 7719 10.1016/elecacta.2008.05.023

[12] Gulaboski R Mirčeski V Bogeski I Hoth M Protein film voltammetry: electrochemical enzymatic spectroscopy. A review on recent progress Journal of Solid State Electrochemistry 2012 16 7 2315 2328 10.1007/s10008-011-1397-5

[13] Laviron E Mounier-Prest R Electrochemical reactions with protonations at equilibrium: Part 14. The cubic scheme Journal of Electroanalytical Chemistry 1992 324 1 18 10.1016/0022-0728(92)80032-Y

[14] Molina A Laborda E Gomez-Gil JM Compton RG Staircase, cyclic and differential voltammetries of the nine-member square scheme at microelectrodes of any geometry with arbitrary chemical stabilization of the three redox states Journal of Solid State Electrochemistry 2016 20 12 3239 3253 10.1007/s10008-016-3308-2

[15] Laborda E Gomez-Gil JM Molina A Microelectrode voltammetry of multi-electron transfers complicated by coupled chemical equilibria: a general theory for the extended square scheme Physical Chemistry Chemical Physics 2017 19 16464 16476 10.1039/C7CP02135F 28608880

[16] Nicholson RS Shain I Experimental verification of an ECE mechanism for the reduction of p-nitrosophenol using stationary electrode polarography Analytical Chemistry 1965 37 2 190 195 10.1021/ac60221a003

[17] Alberts GS Shain I Electrochemical study of kinetics of a chemical reaction coupled between two charge transfer reactions. Potentiostatic reduction of p-nitrosophenol Analytical Chemistry 1963 35 12 1859 1866 10.1021/ac60205a019

[18] Sanecki P Kaczmarski K The voltammetric reduction of some benzenesulfonyl fluorides, simulation of the ECE mechanism and determination of the potential variation of the transfer coefficient by using the compounds with two reducible groups Journal of Electroanalytical Chemistry 1999 471 1 14 25 10.1016/S0022-0728(99)00243-0

[19] Sanecki P Skital P Kaczmarski K Numerical modelling of ECE-ECE and parallel EE-EE mechanisms in cyclic voltammetry. Reduction of 1,4-benzenedisulfonyl difluoride and 1,4-naphthalenedisulfonyl difluoride Electroanalysis 2006 18 10 981 991 10.1002/elan.200603487

[20] Feldberg SW Jeftić Lj Nuances of the ECE mechanism. IV. Theory of cyclic voltammetry and chronoamperometry and the electrochemical reduction of hexacyanochromate(III) The Journal of Physical Chemistry 1972 76 17 2439 2446 10.1021/j100661a017

[21] Wilson GJ Lin CY Webster RD Significant difference in the electrochemical behaviour of the α-, β-, γ- and δ-tocopherols (Vitamin E) The Journal of Physical Chemistry B 2006 110 23 11540 11548 10.1021/jp0604802 16771430

[22] Adams RN Hawley MD Feldberg SW Nuances of the E. C. E. mechanism. II. Addition of hydrochloric acid and amines to electrochemically generated o-benzoquinones The Journal of Physical Chemistry 1967 71 4 851 855 10.1021/j100863a011

[23] Nematollahi D Golabi SM Investigation of the electro-methoxylation reaction: Part 1. Electrochemical study of 4-tert-butylcatechol and 3,4-dihydroxybenzaldehyde in methanol Journal of Electroanalytical Chemistry 2000 481 2 208 214 10.1016/S0022-0728(99)00500-8

[24] Li Y Liu M Xiang C Xie Q Yao S Electrochemical quartz crystal microbalance study of growth and property of the polymer deposit at gold electrode during oxidation of dopamine in aqueous solutions Thin Solid Films 2006 497 270 278 10.1016/j.tsf.2005.10.048

[25] Engblom SO Myland JC Oldham KB Response of an ECE reaction to a potential leap Analytical Chemistry 1994 66 19 3182 3187 10.1021/ac00091a029

[26] Hawley MD Feldberg SW Nuances of the ECE mechanism. I. Development of the theoretical relationship for chronoamperometry The Journal of Physical Chemistry 1966 70 11 3459 3464 10.1021/j100883a015

[27] Galvez J Molina A Saura R Martinez F Dc polarography: current – potential curves with a parallel ECE mechanism: calculation of the rate constants of the chemical reaction Journal of Electroanalytical Chemistry and Interfacial Electrochemistry 1981 127 17 35 10.1016/S0022-0728(81)80464-0

[28] Kastening B Polarographic theory for an ECE [electrochemical – chemical – electrochemical] mechanism Analytical Chemistry 1969 41 8 1142 1144 10.1021/ac60277a016

[29] Sobel HR Smith DE Dc polarography: on the theory for the current – potential profile with an ECE mechanism Journal of Electroanalytical Chemistry and Interfacial Electrochemistry 1970 26 271 284 10.1016/S0022-0728(70)80310-2

[30] Mastragostino M Nadjo L Saveant JM Disproportionation and ECE mechanisms – I. Theoretical analysis. Relationship for linear sweep voltammetry Electrochimica Acta 1968 13 4 721 749 10.1016/0013-4686(68)85007-8

[31] Amatore C Saveant JM Ece and disproportionation: Part V. Stationary state general solution application to linear sweep voltammetry Journal of Electroanalytical Chemistry and Interfacial Electrochemistry 1977 85 1 27 46 10.1016/S0022-0728(77)80150-2

[32] Nicholson RS Shain I Theory of stationary electrode polarography for a chemical reaction coupled between two charge transfers Analytical Chemistry 1965 37 2 178 190 10.1021/ac60221a002

[33] Komorsky-Lovrić Š Lovrić M Theory of staircase cyclic voltammetry of two electrode reactions coupled by a chemical reaction Bulgarian Chemical Communications 2019 51 3 348 357 10.34049/bcc.51.3.4983

[34] Mann MA Helfrick JC Jr Bottomley LA Diagnostic criteria for identifying an ECE mechanism with cyclic square wave voltammetry Journal of the Electrochemical Society 2016 163 4 H3101 H3109

[35] Miles AB Compton RG Simulation of square-wave voltammetry at a channel electrode: E, EC and ECE processes Journal of Electroanalytical Chemistry 2001 499 1 16 10.1016/S0022-0728(00)00460-5

[36] O’Dea JJ Wikiel K Osteryoung J Square-wave voltammetry for ECE mechanisms The Journal of Physical Chemistry 1990 94 9 3628 3636 10.1021/j100372a049

[37] Miles AB Compton RG Simulation of square-wave voltammetry: EC and ECE electrode processes The Journal of Physical Chemistry B 2000 104 22 5331 5342 10.1021/jp0006882

[38] Komorsky-Lovrić Š Lovrić M Theory of square wave voltammetry of two reversible electrode reactions connected by the reversible chemical reaction To Chemistry Journal 2019 2 142 153

[39] Karp S Homogeneous chemical kinetics with rotating disk electrode. ECE mechanism The Journal of Physical Chemistry 1968 72 3 1082 10.1021/j100849a057

[40] Umadevi R Visuvasam J Venugopal K Rajendran L Mathematical models for ECE reactions at rotating disk electrodes using homotopy analysis method AIP Conference Proceedings 2020 2277 130013 1 10.1063/5.0025822

[41] Gulaboski R Surface ECE mechanism in protein film voltammetry – a theoretical study under conditions of square-wave voltammetry Journal of Solid State Electrochemistry 2009 13 1015 1023 10.1007/s10008-008-0665-5

[42] Gulaboski R Kokoškarova P Mitrev S Theoretical aspects of several successive two-step redox mechanisms in protein-film cyclic staircase voltammetry Electrochimica Acta 2012 69 86 96 10.1016/j.electacta.2012.02.086

[43] Strutwolf J Schoeller WW Linear and cyclic sweep voltammetry at a rotating disk electrode. A digital simulation Electroanalysis 1996 8 11 1034 1039 10.1002/elan.1140081111

[44] Lovrić M Modelling electrocatalytic reactions on rotating disk electrodes Russian Journal of Electrochemistry 2022 58 3 202 209 10.1134/S1023193522030077

